# Acute flaccid rhombencephalomyelitis with radiculitis in a child with an enterovirus A71 infection seen for the first time in Denmark: a case report

**DOI:** 10.1186/s13256-021-03246-x

**Published:** 2022-01-25

**Authors:** Pia Jennes Foli-Andersen, Anja Munkholm, Gitte Rønde, Malene Landbo Børresen, Jens Erik Klint Nielsen, Sofie Midgley, Didi Bang

**Affiliations:** 1 Pediatrics Department, Zealand University hospital, Roskilde, Denmark; 2 Pediatric and Adolescent Medicine, Copenhagen University Hospital, Rigshospitalet, Copenhagen, Denmark; 3 Virus and Microbiological Special Diagnostics, Statens Serum Institut, Copenhagen, Denmark; 4 Department of Clinical Microbiology, Copenhagen University Hospital, Amager and Hvidovre, Hvidovre, Denmark

**Keywords:** Acute flaccid myelitis, Enterovirus A71, Limb weakness in children, Severe neurological symptoms, Paralysis, Radiculitis

## Abstract

**Background:**

Acute flaccid myelitis is a serious condition of the spinal cord. More than 80% of patients experience a mild respiratory illness or fever consistent with a viral infection prior to acute flaccid myelitis development. Enterovirus A71 is known to circulate in Denmark, and has previously been associated with severe neurological symptoms. In this case report we describe acute flaccid rhombencephalomyelitis with radiculitis in an infant with an enterovirus infection.

**Case presentation:**

The 8-month-old male of Asian origin presented with fever and gastrointestinal symptoms, followed by severe neurological deficits such as flaccid paralysis of the neck and upper extremities. An initial magnetic resonance imaging scan of the brain was normal, and the boy was treated for encephalitis. A follow-up magnetic resonance imaging scan of the brain and spinal cord 1 week later showed the development of pathological symmetrical gray matter hyperintensity lesions on T2-weighted images in the brainstem and upper medulla spinalis, and nerve enhancement in the terminal thread of the spinal cord and the cervical roots; findings consistent with rhombencephalomyelitis with radiculitis causing flaccid paralysis. Enterovirus A71 was detected in both nasopharyngeal and fecal specimens. Other differential diagnostic etiologies of viral and bacterial encephalitis, including poliovirus, were excluded.

**Conclusions:**

This is the first case in Denmark of a patient diagnosed with acute flaccid rhombencephalomyelitis strongly linked to an enterovirus A71 infection. This case emphasizes the diagnostic importance of combining a history of respiratory and/or gastrointestinal illness, fever, and delayed onset of varying degrees of paralysis with progressive characteristic spinal and brain lesions. Analysis of respiratory, fecal, and cerebrospinal samples for the presence of enterovirus, and eliminating other differential pathogens, is essential to confirm the diagnosis.

## Background

Acute flaccid myelitis (AFM) is a rare condition due to lesions in the gray matter in the spinal cord, leading to paralysis. AFM has a variety of causes including viruses and environmental toxins. In California in 2012, the first cases associating enterovirus (EV) and AFM were reported [1]. During the summer and fall of 2014, a rapid increase in the number of AFM cases was reported in the USA, with no identified common etiology. Simultaneously an unusual clustering of acute limb weakness occurred during a nationwide outbreak of severe respiratory illness among children due to enterovirus D68 (EV-D68). This raised suspicion of a possible association between these neurologic illnesses and EV-D68 infection [1]. In the summer and fall of 2016, the number of AFM cases increased again, coinciding with outbreaks of EV-D68 and enterovirus A71 (EV-A71) [2]. The same pattern repeated in 2018 in the USA [3]. In some cases, EV was detected in nasopharyngeal or fecal specimens, but rarely in the cerebrospinal fluid (CSF). The direct association between EV infection and AFM is still to be determined, and classification remains challenging [4].

## Case presentation

We describe a case of acute flaccid rhombencephalomyelitis (AFREM) in an 8-month-old male Indian infant living in Denmark. The patient was born at term, previously healthy with appropriate motor and psychological development, and fully vaccinated according to Danish standards. In August 2018, 5 days after traveling from India to Denmark, the patient was hospitalized at a regional hospital in Denmark. He presented with fever (37.5–38.5 °C), stomach pain, vomiting, and a couple of loose stools. Two days prior to admission, increasing drowsiness and debilitation occurred. There was no history of head trauma or seizures, and no history of intoxication. On admission, there was intermittent loss of consciousness, nystagmus, and generalized muscular hypotonia. The patient had difficulty holding his head upright, and this further developed into almost complete paralysis of the upper extremities. All vital parameters were normal, but due to breathing difficulties the patient was transferred to intensive care at the referral hospital. An urgent computed tomography (CT) of the brain was normal. Initial laboratory evaluation including white blood cell count, hemoglobin, electrolytes, and liver parameters were all normal. Treatment with ceftriaxone 100 mg/kg/daily and acyclovir 20 mg/kg*3/daily for suspected encephalitis was initiated. The CSF examination revealed a white blood cell count of 27 cells/mm^3^ (≤ 5 cells/mm^3^), predominantly lymphocytes (74%), a protein concentration of 0.38 g/L (0.4–0.7 g/L), and a normal glucose concentration. CSF tested negative for bacteria by culture, and negative for *Mycoplasma pneumoniae*, *Chlamydophilia pneumonia*, *Legionella* sp., *Neisseria meningitides*, *Haemophilus influenzae*, *Listeria monocytogenes*, *Streptococcus pneumoniae*, *Streptococcus agalactiae*, *Cryptococcus neoformans*, *Escherichia coli* (K1), enterovirus species A–D, herpes simplex virus, varicella zoster virus, cytomegalovirus, and human herpes virus 6 A/B by PCR. The nasopharyngeal specimen was PCR positive for enterovirus, rhinovirus, and *Mycoplasma pneumoniae*, whereas PCRs for influenza A/B, respiratory syncytial virus, human metapneumovirus, parainfluenzavirus (1–4), coronavirus (229E, HKU1, OC43, NL63), adenovirus, *Chlamydophila pneumoniae*, and *Bordatella pertusis* were negative. Ciprofloxacin 3 mg/kg*3/daily was added to treat a suspected mycoplasma encephaloradiculitis.

**Fig. 1:**
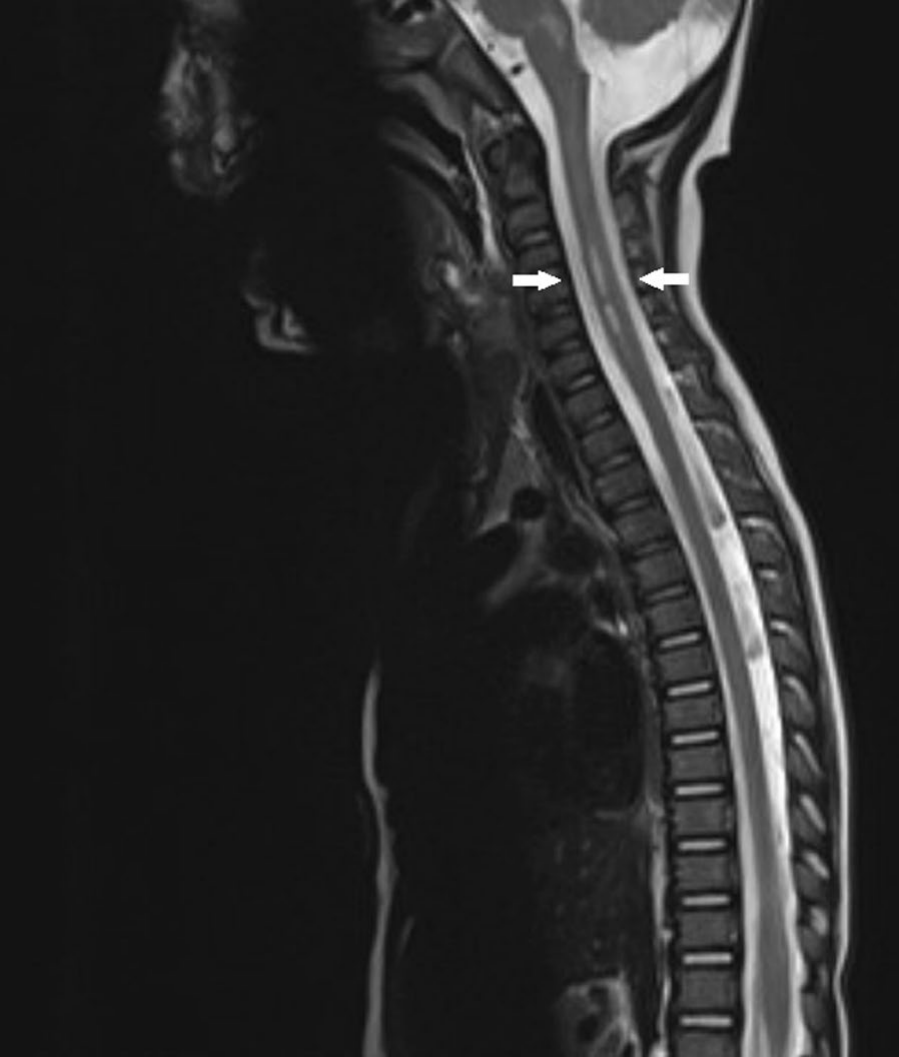
Sagittal T2-weighted magnetic resonance imaging of the spinal cord 1 week after onset of neurological symptoms demonstrated a longitudinal spinal cord lesion of the gray matter at the cervical level C3–C5. See arrows. These findings were consistent with rhombencephalomyelitis with radiculitis

The fecal sample was PCR positive for enterovirus, and PCR negative for norovirus, rotavirus, adenovirus, astrovirus, and sapovirus. Intestinal pathogen PCRs for *Salmonella*, *Shigella*, *Campylobacter*, *Yersinia*, *Vibrio*, *Aeromonas* sp., and *Clostridium difficile* were negative. Cell culture for poliovirus was negative. The differential diagnosis considered was acute disseminated encephalomyelitis. The electroencephalogram (EEG) was normal and an initial brain magnetic resonance imaging (MRI) was normal. The condition was treated as encephalomyelitis, and treatment with intravenous immunoglobulin (IG) 2 g/kg and methylprednisolone 20 mg/kg was initiated 1 day after admission. A follow-up MRI of the brain and the spinal cord 1 week later showed development of pathological symmetrical signal changes of the gray matter of the medulla oblongata on T2-weighted images resembling rhomboencephalitis, as well as similar changes in the cervical spinal cord at C3–C5. There was enhancement of the cervical roots and terminal thread of the spinal cord. See Fig. 1.

Enterovirus A71 was detected both in the nasopharyngeal and fecal specimens, but not in the CSF. Analysis of partial VP2 sequences revealed a subtype of EV-A71 known to circulate in Denmark and other parts of the world, and previously detected in cases of rhombencephalitis and AFM [2, 5, 6]. The infant received intensive physiotherapy from an early stage, and follow-up visits showed full recovery within 2 weeks. The extent of recovery was assessed by the physiotherapist and the parents.

## Discussion and conclusions

To our knowledge, this is the first case in Denmark diagnosed with AFREM with detection of EV-A71 in fecal and nasopharyngeal samples. The definition of AFM is acute onset of flaccid limb weakness and spinal MRI lesions largely restricted to spinal gray matter spanning over one or more spinal segments.

Approximately 80% of children with AFM experience respiratory or gastrointestinal symptoms, and 75% have fever days prior to paralysis. Onset of paralysis is generally abrupt, with rapid progression within a few days [7]. Paralysis is often asymmetric monoplegia, but quadriplegia has been described [4]. As in our case, upper limb paralysis is most common [8].

AFM should be suspected in a patient with onset of acute flaccid limb weakness with decreased reflexes in contrast to demyelinating white matter lesions in the spinal cord, which cause hypertonia and hyperreflexia. If the MRI of the spinal cord shows typical T2 hyperintensity gray matter (2. Neuron) lesions, the diagnosis is strongly suspected. These findings are the hallmark of AFM [9]. As in our case, the spinal cord lesions may not be present on an initial MRI. A normal MRI performed within the first 72 hours after onset of limb weakness does therefore not rule out AFM, and a repeat MRI is recommended. In most cases, mild to moderate pleocytosis (usually < 100 cells/mm^3^) with lymphocytic predominance is present in the acute phase. The MRI findings of AFM differ from other diseases in the spinal cord with similar symptoms. Other etiologies of acute limb weakness such as central nervous infections with herpes simplex virus, varicella zoster virus, cytomegalovirus and bacteria resulting in Guillain–Barre syndrome and cerebral hemorrhage should also be considered.

The annual rate of acute flaccid paralysis among children under 15 years of age is expected to occur in 1 per 100,000 children [10]. The most consistent theory is that AFM has a viral etiology, rather than other infectious pathogens or autoimmune disorders. Different viruses have been associated with AFM, such as non-polio enteroviruses (EV), adenoviruses, and West Nile virus. Since viruses are rarely isolated in the CSF, the association has been difficult to confirm [8, 11, 12] However, recent studies on EV-D68 and EV-A71 appear to support this association [1, 6, 13, 14].

The potential link between EV and AFM is based on the finding of EV in fecal and/or nasopharyngeal samples, the outbreaks of EV and AFM overlapping in time, and the finding in mouse studies that EV can lead to CNS damage [15].

In our case, the findings of EV-A71, a pathogen known to be associated with AFM, in both nasopharyngeal and fecal samples highly suggests the AFREM association. It is not unusual not to find EV-A71 in spinal fluid, as seen in outbreaks of enterovirus-linked neurological diseases in Catalonia (2016), France (2016), and the USA (2018) [6, 12, 16]. CSF samples were only positive for EV in 11% (Catalonia), 13.5% (France), and 20% (USA). In EV-CSF negative cases, EV was detected in fecal and/or nasopharyngeal samples. The World Health Organization (WHO) refers to detection rates of EV-A71 from CSF samples as being less than 5%. The difference in detection rate may depend on the timepoint during the infection when the CSF was tapped. The lack of detection of EV in CSF could be because the virus is inside the CNS parenchyma, or because the virus has already been cleared from the CNS [15].

A causality study in the USA tested EV-D68 antibodies in the CSF of patients with AFM and EV confirmed in fecal and/or nasopharyngeal samples [15]. Direct PCR detection of EV-D68 was found in 1 of 14 samples, whereas indirect EV-D68 antibodies were found in 11 of 14 samples. This was significantly higher than controls, and the study provides evidence that EV may be a causal factor in the development of AFM. Although interpretation of EV antibodies may pose difficulties due to cross reactivity. More studies are needed in this area.

It is not possible to determine conclusively whether the infection was contracted in Denmark or India before the patient’s arrival 5 days later in Denmark, as the incubation period varies from 3 to 10 days and the onset of neurological symptoms up to 10 days later. There were a limited number of reported infections with EV-A71 of the same subtype during the period of diagnosis for the patient presented in this case report (unpublished data, Sofie Midgley SSI). We were not aware of any ongoing epidemic with EV-A71 in India at the time.

Management of children with AFM follows basic standards of care for children with severe neurologic disease. No evidence of definitive efficacy exists in specific treatments of AFM using corticosteroids, intravenous IG, plasmapheresis, interferon, antivirals, or other immunomodulatory agents. However, often corticosteroids and intravenous IG are initiated as in our case, based on the patients clinical presentation [7]. Physical therapy implemented as soon as the child is stable may optimize functional outcomes, as seen in this case.

Enteroviruses are very common viruses, with an unknown prevalence. Enterovirus infections are most commonly asymptomatic or present as mild illness, often with respiratory or gastrointestinal symptoms. Severe cases including myocarditis, meningitis, and encephalitis are rare [16]. Most severe cases have been observed in young children [2, 7, 10]. It is still unclear why paralysis occurs in a very small minority of infected children. The European non-polio enterovirus network (ENPEN) recommend that cerebrospinal fluid, nasopharyngeal, and fecal samples be sent for enterovirus testing from patients with possible neurological infections [17]. In this case, EV-A71 was found in both fecal and nasopharyngeal specimens, but not the CSF.

This case report presents a case of rhombencephalomyelitis with radiculitis, with a strong link to an EV-A71 infection. The awareness of EV infections as a cause of neurological illness needs to be emphasized, in order to identify these cases. Surveillance of infections with these enterovirus subtypes is necessary, and further investigation to understand the pathogenic association of specific subtypes with severe neurological disease is warranted.

## Data Availability

All data generated or analyzed during this study are included in this published article.

## Abbreviations

AFM: Acute flaccid myelitis
CT: Computed tomography
CSF: Cerebrospinal fluid
EV: Enterovirus
MRI: Magnetic resonance imaging
